# APC/β-catenin-rich complexes at membrane protrusions regulate mammary tumor cell migration and mesenchymal morphology

**DOI:** 10.1186/1471-2407-13-12

**Published:** 2013-01-09

**Authors:** Matthew A Odenwald, Jenifer R Prosperi, Kathleen H Goss

**Affiliations:** 1 Department of Surgery, University of Chicago, Chicago, IL, 60637, USA; 2 Current Address: Department of Biochemistry and Molecular Biology, Indiana University School of Medicine – South Bend, South Bend, IN, 46617, USA

## Abstract

**Background:**

The APC tumor suppressor is mutated or downregulated in many tumor types, and is prominently localized to punctate clusters at protrusion tips in migratory cells, such as in astrocytes where it has been implicated in directed cell motility. Although APC loss is considered an initiating event in colorectal cancer, for example, it is less clear what role APC plays in tumor cell motility and whether loss of APC might be an important promoter of tumor progression in addition to initiation.

**Methods:**

The localization of APC and β-catenin was analyzed in multiple cell lines, including non-transformed epithelial lines treated with a proteasome inhibitor or TGFβ to induce an epithelial-to-mesenchymal transition (EMT), as well as several breast cancer lines, by immunofluorescence. APC expression was knocked down in 4T07 mammary tumor cells using lentiviral-mediated delivery of APC-specific short-hairpin (sh) RNAs, and assessed using quantitative (q) reverse-transcriptase (RT)-PCR and western blotting. Tumor cell motility was analyzed by performing wound-filling assays, and morphology via immunofluorescence (IF) and phase-contrast microscopy. Additionally, proliferation was measured using BrdU incorporation, and TCF reporter assays were performed to determine β-catenin/TCF-mediated transcriptional activity.

**Results:**

APC/β-catenin-rich complexes were observed at protrusion ends of migratory epithelial cells treated with a proteasome inhibitor or when EMT has been induced and in tumor cells with a mesenchymal, spindle-like morphology. 4T07 tumor cells with reduced APC levels were significantly less motile and had a more rounded morphology; yet, they did not differ significantly in proliferation or β-catenin/TCF transcriptional activity. Furthermore, we found that APC/β-catenin-rich complexes at protrusion ends were dependent upon an intact microtubule cytoskeleton.

**Conclusions:**

These findings indicate that membrane protrusions with APC/β-catenin-containing puncta control the migratory potential and mesenchymal morphology of mammary tumor cells and suggest that APC loss during later stages of tumor progression might impact tumor cell dissemination or colonization.

## Background

The *adenomatous polyposis coli* (*APC*) tumor suppressor gene was initially identified as the gene mutated in familial adenomatous polyposis (FAP), a rare inherited colorectal cancer syndrome that predisposes carriers to hundreds to thousands of adenomatous polyps and aggressive, early-onset colon cancer [1-3]. Identification of *APC* mutations and loss of heterozygosity (LOH) in approximately half of sporadic colorectal adenomas and majority of adenocarcinomas indicates that *APC* inactivation is an early event in tumor progression (i.e. initiation). It is now appreciated that *APC* mutation is relatively rare in other solid tumors but it is silenced by gene methylation in multiple tumor types, including breast cancer (reviewed in [4]). Recent studies from our laboratory demonstrated that APC is required for maintaining epithelial homeostasis in the mouse mammary gland and its mutation cooperates with other oncogenic alterations, such as overexpression of the Polyoma Middle T antigen (PyMT) oncogene [5,6], to enhance primary mammary tumorigenesis. These studies have indicated that the effects of *Apc* mutation in both normal mammary homeostasis and mammary tumor development are not solely dependent on Wnt pathway regulation, which is APC’s best-characterized molecular activity. Through de-repression of the canonical Wnt pathway, APC loss results in β-catenin stabilization, accumulation and nuclear translocation where it associates with TCF/LEF transcription factors to regulate Wnt target genes. In addition to inducing tumor cell proliferation, Wnt target genes also promote cell survival, stem cell self-renewal, and matrix remodeling [7]. Yet, emerging data like those in the mammary models described above indicate that the consequences of APC inactivation are not solely restricted to Wnt pathway activation.

Among the functions that have been attributed to APC independent of regulating Wnt signaling are mediating genomic stability, apoptosis, DNA repair, proliferation, and apical-basolateral and front-rear polarity (reviewed in [8,9]). Robust microtubule-dependent localization of endogenous and overexpressed APC to cell protrusion ends in migrating cells [10-13] suggests that this major pool of APC is likely to be involved in guiding front-rear cell polarity and motility. In fact, APC is required for directed motility of astrocytes [14,15]. Despite these findings, it is not clear how this pool of APC, and its role at the leading edge and at membrane protrusions in migration, contributes to tumor suppression mediated by APC and drives tumor initiation or progression when *APC* is mutated. In the current study, we have characterized APC/β-catenin complexes at membrane protrusions in epithelial cells undergoing an epithelial-to-mesenchymal transition (EMT) and in human and mouse breast cancer cells. Using an *in vitro* model of APC depletion in the 4T07 mouse mammary tumor cells, we demonstrate that disruption of these complexes inhibits tumor cell migration and disrupt mesenchymal cell morphology. These results indicate that invasive tumor cell behavior is dependent on APC/β-catenin complexes at membrane extensions and suggests that perturbation of these complexes in motile tumor cells via APC inactivation may significantly impact tumor cell dissemination and colonization.

## Methods

### Cell culture

EpH4 mouse mammary epithelial cells [16] and Madin-Darby Canine Kidney (MDCK) were obtained from Karl Matlin ([17]; University of Chicago) and maintained at 37°C with 5% CO_2_ in DMEM supplemented with 5% fetal bovine serum (FBS), 1% penicillin/streptomycin, and 1:5000 Plasmocin (Invivogen). 4T1, 4T07, and 67NR mouse mammary tumor cells obtained from Fred Miller ([18]; Karmanos Cancer Institute), and Hs578T human breast cancer and SW480 colon cancer cells obtained from the American Type Culture Collection (ATCC) were maintained in DMEM with 10% FBS and pen/strep as above. HCT116 colon cancer cells (ATCC) were maintained in McCoy’s media with 10% FBS. Selection agents were added as described below. All cells were passaged every 2–3 days to maintain exponential growth. Subconfluent MDCK and EpH4 cells were incubated with N-Acetyl-Leu-Leu-Nle-CHO (ALLN; Calbiochem) for 24 h at the indicated concentrations. Nocodazole (10 μg/ml; Sigma) was added to the culture medium for the times indicated. Control cells were treated with DMSO at identical concentrations as drug-treated cells. For EMT experiments, MDCK cells were treated with 10 ng/ml human recombinant TGFβ (R&D) or ethanol for 24 h. Membrane protrusions were counted from phalloidin-stained slides without knowledge of the treatment and classified as having zero to one, two to four, or more than four processes. At least 100 cells were counted per treatment, and the experiments were performed in triplicate, and statistical analyses were performed using the Student’s t-test. In 4T07 APC knockdown experiments, morphology was quantified from phalloidin-stained fixed cells (at least five representative fields per cell line per experiment) without knowledge of the cell line identity, and cells were classified as having two protrusions (bipolar) or a rounded morphology. Statistical analysis was performed using the Fisher’s exact test.

### Western blotting

For β-catenin immunoblotting, total cell lysates of ALLN-treated cells were separated on a 10% SDS-PAGE gel, and transferred to Immobilon P membrane (Millipore). The blots were probed with anti-β-catenin monoclonal antibody (BD Biosciences) at a 1:1000 dilution and stripped and reprobed with anti-actin mouse monoclonal antibody (Sigma; 1:2000 dilution). For APC western blotting, total lysates were extracted with 50 mM Tris pH7.5, 0.1. IGEPAL, 100 mM NaCl, 1 mM MgCl_2_, 5 mM EDTA and protease inhibitors and sonicated. 50 μg of lysates from each cell line were separated on a 7% SDS-PAGE gel and transferred to Immobilon P membrane and probed with 1:3000 anti-APC polyclonal antibody [19] obtained from Kristi Neufeld (University of Kansas) in blocking buffer (5% nonfat dried milk in TBST). HCT116 lysates were used as a control for full-length APC. Densitometry was performed using Image J software (NIH).

### Generation of APC-knockdown cell lines

Prior to generating stable APC-knockdown cell lines, 4T07 cells were stably transfected with the green fluorescent protein (GFP)-containing vector pSEB-HUS (obtained from Tong-Chuan He, University of Chicago), and selected using 5 μg/ml blasticidin (Invitrogen). For stable APC-knockdown experiments, 4T07-GFP cells were infected with one of two different shAPC lentiviral constructs (Sigma Aldrich), and selected using 1.5 μg/ml puromycin (Sigma). For controls, the 4T07-GFP cell line was infected with an empty vector (pLKO.1) or the SHC-002 scrambled vector (Sigma). β-catenin/TCF luciferase reporter assays in these cells were performed as described previously [6], and SW480 cells were used as a positive control. For transient knockdown experiments, 2 × 10^6^ 4T07 cells were electroporated with 1.5 mg siRNA against *Apc* (Ambion) or a nonsense control siRNA (Ambion) using the Nucleofector Kit V following the manufacturer's protocol on setting D-032 on a Nucleofector electroporator (Amaxa- and plated on glass coverslips in 12-well plates. For transient knockdown of β-catenin, 4.5 × 10^4^ MDCK cells were transfected with 30 pmol either β-catenin siRNA (Dharmacon) or negative control siRNA (Ambion) using RNAiMAX (Invitrogen) according to the manufacturer’s recommendations. After 24 h, transfected cells were treated with 15uM ALLN or the vehicle (DMSO) for 24 h, at which point cells were fixed and stained as below.

### Quantitative Reverse-Transcriptase PCR (qRT-PCR)

RNA was isolated using TRI Reagent (Molecular Research Center). 5 μg of total RNA was used to generate cDNA using Super Script III Reverse Transcriptase (Invitrogen). *Apc* was amplified by qPCR using Power SYBR Green mix (Invitrogen) and the Opticon 2 real-time PCR machine (Bio-Rad) and normalized to 18S levels as described [5]. The following primers were used for either *Apc* or *18S*: *Apc,* forward 5^′^- AGG CAG AGT CCC TCA CAG AA-3^′^ and reverse 5^′^- CAC TGG TTC CCC TTG ACC TA-3^′^; and *18S*, forward 5^′^-GGC GGC TTG GTG ACT CTA GAT-3^′^ and reverse 5^′^-CTT CCT TGG ATG TGG TAG CCG-3^′^. Experimental duplicates were analyzed, and normalized values were averaged.

### Immunofluorescence

Cells were grown to approximately ~70% confluence on glass coverslips, fixed with 3.7% paraformaldehyde for 15 min and permeabilized with 0.3% Triton-X100 in PBS for 5 min. The following primary antibodies were diluted in 5 mg/ml bovine serum albumin (BSA) and 0.5% NP-40 in PBS at 37°C: anti-APC 1:400 (rabbit polyclonal, provided by Inke Näthke at the University of Dundee; [10]), anti-β-catenin 1:400 (mouse monoclonal, BD Biosciences) or 1:100 (rabbit polyclonal, Lab Vision Corporation), and anti-E-cadherin 1:100 (mouse monoclonal, BD Biosciences). Staining was visualized with either with FITC-, Rhodamine- (Pierce) or Alexa-conjugated goat anti-mouse or -rabbit IgG (Invitrogen) in BSA/NP-40/PBS. Alexa Fluor 488- or 594- conjugated phalloidin (1:200, Invitrogen) was used to visualize the actin cytoskeleton. Cells were counterstained with Hoechst nuclear dye (Sigma), mounted with Fluoromount G (Electron Microscopy Sciences) and analyzed on a Zeiss Axioskop 2 Plus upright fluorescent microscope using a Plan-Apochromat 63x/1.4 oil objective. Images were captured with Axiocam MRm digital monochrome camera and Axiovision 3.1 or 4.6 imaging software (Zeiss).

### BrdU incorporation assay

4 x 10^4^ cells were plated in triplicate on coverslips in 12-well plates, and after 24 h, 10 μM 5-bromo-2'-deoxyuridine (BrdU; BD Biosciences) diluted in growth media was added to each well. The cells were fixed in 3.7% formaldehyde 12 h after BrdU addition. DNA was denatured with 2 M HCl for 20 min at 37°C, and this reaction was neutralized in a borate buffer (pH 9.0) for 1 min at room temperature. Antigenic sites were exposed by incubation in trypsin solution (0.005% trypsin in 0.05 M Tris pH 8.3) at 37°C. After blocking as above, a 1:400 dilution of anti-BrdU (rat monoclonal, Abcam) in blocking buffer was added to each well and incubated at 37°C for 1 h. Staining was visualized with a 1:200 dilution of rhodamine-conjugated anti-rat antibody (Pierce) and the coverslips were mounted and analyzed as above. Quantification was determined by counting the number of BrdU-positive nuclei out of at least 100 cells per well; three independent experiments were performed in triplicate.

### Wound-filling assay

4T07 stable APC-knockdown and control cells were plated in triplicate at a density of 1 x 10^5^ cells per well in a 6-well plate and grown to confluence at which time 10 μg/mL mitomycin C (Sigma) was added to inhibit proliferation. Three scratch wounds were made in each well with a 0.10-10-μL pipette tip, and mitomycin C-containing media was changed every 24 h. Phase-contrast images were acquired using an Axiovert 35 inverted microscope (Zeiss) and AxioVision REL 4.6 imaging software (Zeiss) at 0, 12, 24, and 36 h post-wounding. The area of three representative wounds of each cell line was quantified and averaged at each time point using ImageJ software.

### Statistical analysis

All experiments were performed at least in triplicate, and statistical significance was defined as a p value < 0.05 using a one-way ANOVA with a Student’s t-test post-test, unless noted otherwise. For quantification, means are shown with standard deviation.

## Results

**Figure 1:**
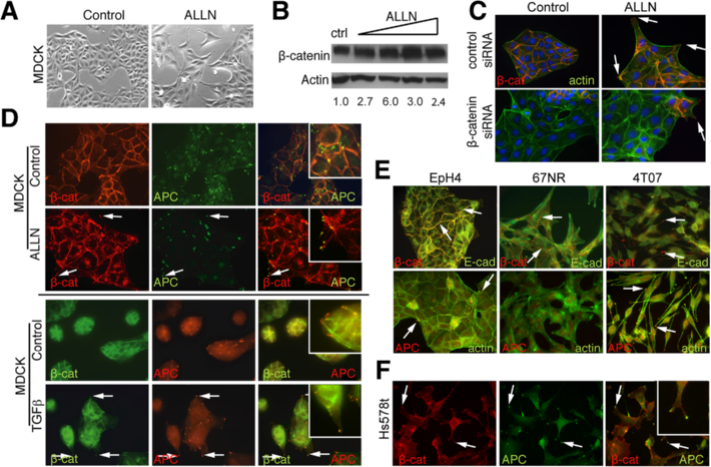
**Characterization of APC/β-catenin-rich clusters at membrane protrusion ends.** (**A**) MDCK cells were treated for 24 h with 15 μM ALLN or DMSO, and phase-contrast microscopy demonstrated cell flattening and extensive membrane protrusions in drug-treated cells. 400X. (**B**) Total cell lysates were with anti-β-catenin and -β-actin antibodies. There was an increase in total β-catenin ALLN (range 0.5-30 μM); and the values shown represent densitometry of β-catenin normalized to actin relative to control cells. (**C**) MDCK cells were transfected with β-catenin or non-targeting siRNAs and treated with 15 μM ALLN or DMSO for 24 h, and stained with anti-β-catenin (red) or phalloidin (green). With β-catenin knockdown, there are very few protrusions with ALLN compared to adjacent areas in which the cells are β-cateninproficient (arrows). 630X. (**D**) MDCK cells were treated with 15 μM ALLN or DMSO (top) or 10 ng/ml TGFβ or ethanol for 24 h (bottom). The cells were stained with anti-β-catenin (red) and -APC (green) antibodies; arrows indicate co-localization of β-catenin and APC at membrane protrusions. 630X. (**E**) EpH4 and 67NR and 4T07 cells were stained with anti-β-catenin (red), –E-cadherin (green), and –APC (red) antibodies and phalloidin. Top, arrows indicate β-catenin localization to the lateral membrane in EpH4 and 67NR cells but to protrusions in 4T07 cells. Bottom, arrows indicate punctate APC localization at the basal surface in EpH4 and at membrane protrusions in 4T07 cells. 630X. (**F**) Hs587t human breast cancer cells show β-catenin (red) and APC (green) co-localization of membrane extension tips. 630X.

To examine whether endogenous β-catenin localized to membrane protrusions like APC [10] and exogenous mutant β-catenin [20], β-catenin was stabilized with the proteasome inhibitor ALLN in MDCK kidney epithelial cells. ALLN treatment resulted in altered cell morphology compared to vehicle controls; specifically, treated cells appeared flatter and more spread and had abundant long, thin membrane processes (Figure 1A). Treatment with the proteasome inhibitor also significantly increased expression of total β-catenin protein by western blotting over a range of concentrations (Figure 1B). To further implicate β-catenin in these phenotypes, β-catenin was knocked down in MDCK cells using siRNAs. Staining of the cells with phalloidin and anti-β-catenin antibody by IF confirmed that the compact epithelial morphology and tight cell-cell interactions of MDCK cells were perturbed by β-catenin silencing in the absence of ALLN (Figure 1C). β-catenin down-regulation abrogated the effect of ALLN on protrusion formation compared to a non-targeting control siRNA (Figure 1C).

To characterize the cellular processes that formed following stabilization of endogenous β-catenin, IF for β-catenin was performed on ALLN-treated MDCK cells. In control cells, the localization of β-catenin was restricted to the membrane at sites of cell-cell contact as expected, but stabilized endogenous β-catenin was at the membrane, heterogeneous in nuclei, and, unexpectedly, prominently localized at the tips of long membrane protrusions in ALLN-treated cells (Figure 1D). Consistent with previous observations [10,21], APC was localized to the leading edge and in clusters at the ends of membrane protrusions of subconfluent MDCK cells (Figure 1D). In cells treated with ALLN, β-catenin co-localized with APC at the tips of membrane protrusions (Figure 1D). To explore whether there were other circumstances in which β-catenin co-localized with APC at protrusion ends in epithelial cells, we analyzed the well-characterized model of TGFβ-induced EMT in MDCK cells. Unlike untreated MDCK cells, upon TGFβ exposure, β-catenin localization was evident at the tips of membrane extensions, where it co-localized with APC (Figure 1E). These data indicate that in epithelial cells in which endogenous β-catenin is stabilized or that are undergoing EMT, β-catenin shifts from an exclusively membrane-associated localization to an additional complex at the ends of membrane protrusions where it co-localizes with the APC tumor suppressor.

β-catenin stabilization and subsequent Wnt pathway activation, as well as EMT, are associated with the progression of many tumor types, including breast cancer. Therefore, we next explored whether APC/β-catenin complexes at protrusion ends were found in breast cancer cells. For this, we turned to a progression series set of mouse mammary cell lines, all derived from the same genetic background, that range from non-transformed (EpH4) to tumorigenic but non-metastatic (67NR) to highly invasive (4T07) [18]. Like MDCK cells, nontransformed EpH4 mouse mammary epithelial cells have β-catenin restricted to the lateral membrane at cell-cell contacts and co-localized with E-cadherin, whereas, APC is in a punctate localization pattern at the few membrane protrusions these cells have (Figure 1E). The membrane-associated pool of β-catenin was maintained in 67NR cells, and compared to EpH4, these cells had a more elongated appearance with decreased cell-cell interactions and obvious membrane protrusions; yet, neither β-catenin nor APC was associated with these protrusions (Figure 1E). 4T07 cells exhibited a spindle-like morphology and lacked obvious cell-cell contacts with E-cadherin or β-catenin membrane localization. However, β-catenin and APC were prominently localized at the ends of the long membrane protrusions in 4T07 cells (Figure 1E). Human breast cancer cells, including the ER-negative Hs578T line, showed the identical pattern of APC/β-catenin co-localization at protrusion ends (Figure 1F).

**Figure 2:**
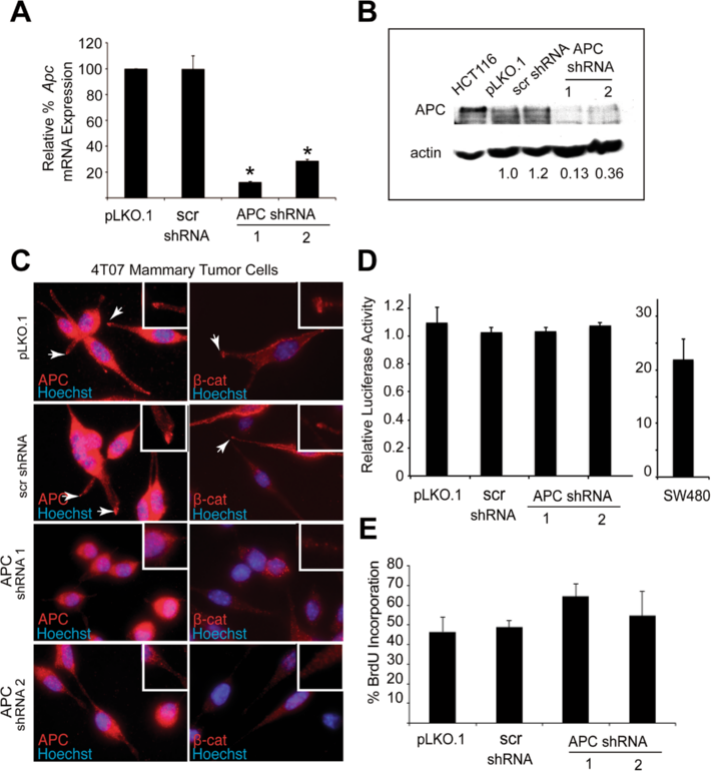
**APC knockdown in 4T07 cells perturbs β-catenin localization at protrusion ends but does not induce β-catenin/TCF-transcriptional activity.** (**A**) *Apc* mRNA expression was analyzed in 4T07 stable pools expressing vector only (pLKO.1), a scrambled (scr) shRNA and two independent APC-specific shRNAs by qRT-PCR. Values were normalized to 18S rRNA expression, and data are presented relative to expression in pLKO.1 control cells, set at 100% (*p < 0.05 compared to pLKO.1). (**B**) Lysates from 4T07 control and APC-knockdown cells were subjected to western blotting with an anti-APC antibody [19]. Lysates from HCT116 cells are included as a control for full-length APC. Densitometry values are APC levels normalized to actin relative to the pLKO.1 control cells. (**C**) IF with anti-APC and -β-catenin antibodies illustrate co-localization to membrane protrusion ends. In contrast, APC-knockdown cells lack specific localization of APC or β-catenin to protrusion ends and generally have a more rounded morphology. Images were taken at the same exposure level for APC protein; cytosolic staining is non-specific. 630X. (**D**) Cells were transfected with pTOPFlash containing TCF consensus sites upstream of luciferase or pFOPflash with mutant TCF-binding sites. Transfection efficiency was normalized by transfection of pRL-TK, and the data are plotted as the TOPflash/FOPflash ratio. SW480 cells are a positive control, and there are no significant differences between APC-knockdown and control 4T07 cells. (**E**) BrdU incorporation assays were performed on APC-knockdown and control cells, and the percentage of cells with BrdU incorporation after 24 h was quantified by IF with an anti-BrdU antibody and plotted.

To determine the importance of APC/β-catenin-containing complexes at the protrusions in mammary tumor cell behavior, we stably suppressed APC expression using lentiviral-mediated expression of APC shRNAs. We first introduced GFP into the 4T07 cells for visualization and then generated stable 4T07 pools that expressed one of two different APC-specific shRNAs, a scrambled shRNA (SHC-002) or empty vector (pLKO.1). The expression of APC mRNA in the control and APC shRNA-infected cell lines was analyzed by qRT-PCR, and we found that APC expression was significantly reduced by 70-90% in the APC shRNA-infected cells relative to the pLKO.1-infected cells (Figure 2A; p < 0.01). Total cell lysates from each cell line were analyzed by western blotting with an APC antibody and show that there is a significant reduction in full-length APC protein expression upon knockdown (64-87% depending on the construct) (Figure 2B), consistent with the mRNA levels. The cells were then analyzed for expression and localization of APC protein by IF (Figure 2C). Both APC shRNA cell lines had a lower intensity of staining compared with the control lines, and the specific localization of APC at cell protrusion ends was absent. Additionally, the spindle-shaped morphology of the cells was attenuated, and the cells displayed a slightly more rounded shape. Suppression of APC expression and the morphological change was concomitant with a loss of β-catenin also at protrusion ends (Figure 2C). Despite the fact that APC loss is often associated with β-catenin stabilization and nuclear accumulation, this was not observed in the APC-knockdown 4T07 cells, either by IF or western blotting (Figure 2C and data not shown). Consistently, there was no induction of β-catenin/TCF-mediated transcriptional activity as determined by a TOPflash reporter assay (Figure 2D). These data are also consistent with our previous observations that heterozygous *Apc* mutation (*in vivo*) or APC knockdown (*in vitro*) was insufficient for hyperactivation of canonical Wnt signaling in mammary epithelial cells [5]. Moreover, there was not a statistically significant difference in BrdU incorporation, a measure of S-phase entry, between control and APC-knockdown cell lines, although there was a trend to increased proliferation in the APC shRNA1 cells (Figure 2E). Since APC has been shown to control the G_1_/S transition by regulating the β-catenin/TCF transcriptional targets c-myc and cyclin D1 [22], these results suggest that APC suppression in these invasive breast cancer cells impacts neither β-catenin-mediated transcription nor proliferation.

**Figure 3:**
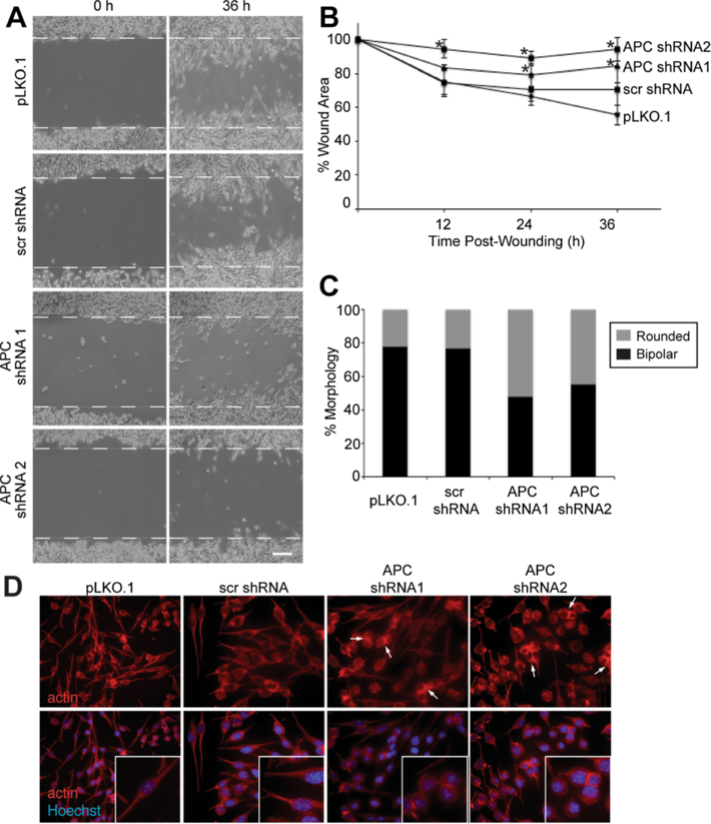
**APC is required for mammary tumor cell migration and mesenchymal morphology.** (**A**) Wound-filling assays were performed by scratching confluent cultures of APC–knockdown and control 4T07 cells in the presence of 10 μg/ml mitomycin c and capturing phase-contrast images at 0, 12, 24, and 36 h post-wounding. Representative images from 0 and 36 h are shown; dashed white lines represent location of the wound. APC-knockdown cells were impaired in their ability to fill the wound at 36 h. 100X. (**B**) Quantification of the wound area at all time points shows that APC knockdown significantly decreased the migration of 4T07 cells at all time points. *p < 0.05 compared to pLKO.1 cells. (**C**) The morphology of APC-knockdown cells was quantified on phalloidin-stained cells grown on coverslips and categorized as having two protrusions (bipolar) or a rounded morphology. APC-knockdown cells have a higher percentage of cells with a rounded morphology compared to controls (p < 0.05 for APC-knockdown cells vs. pLKO.1 cells by Fisher’s exact test). (**D**) Representative images of the cells whose morphology was quantified in (C) and stained with phalloidin (red) and Hoechst (blue). Arrows illustrate cell-cell interactions, which are more prominent in APC-knockdown cells. 630X.

The localization of APC/β-catenin complexes to protrusion ends and the subtle disruption of the spindle-shaped morphology of 4T07 cells with APC knockdown suggests that these complexes may be important in tumor cell motility and invasion, particularly in response to a stimulus such as wounding or growth factors/cytokine signaling. Wound-filling assays were performed in the presence of the cell cycle inhibitor mitomycin C in control and APC-knockdown cells. Images were acquired over the time-course (up to 36 h), and representative phase-contrast images are shown, demonstrating that the cells with APC knockdown had an impaired ability to fill in the wound compared to control cells (Figure 3A). Control cells at the wound edge had a spindle-shaped morphology with long membrane protrusions, but very few of the APC-knockdown cells had this morphology. Quantification of the unfilled area of the wound at each time point (0, 12, 24, and 36 h post-wounding) indicated that there was a statistically significant larger wound area in the APC-knockdown cell lines at all time points compared to the vector-infected control cells (Figure 3B). Because of this effect and the morphological changes in the APC-knockdown cells noted earlier, we examined tumor cell morphology more thoroughly. Control and APC-knockdown 4T07 cells were plated at subconfluent density and stained with phalloidin so that cell protrusions could be readily visualized. The morphology of the cells was characterized as having two protrusions (bipolar), typical of parental 4T07 cells (Figure 1E), or rounded. With knockdown observed a significant decrease in the bipolar morphology and concomitant increase in the rounded phenotype (Figure 3C-D). Additionally, more frequent cell-cell contacts and generally blunted cell protrusions were observed in the cells with APC knockdown (Figure 3D).

**Figure 4:**
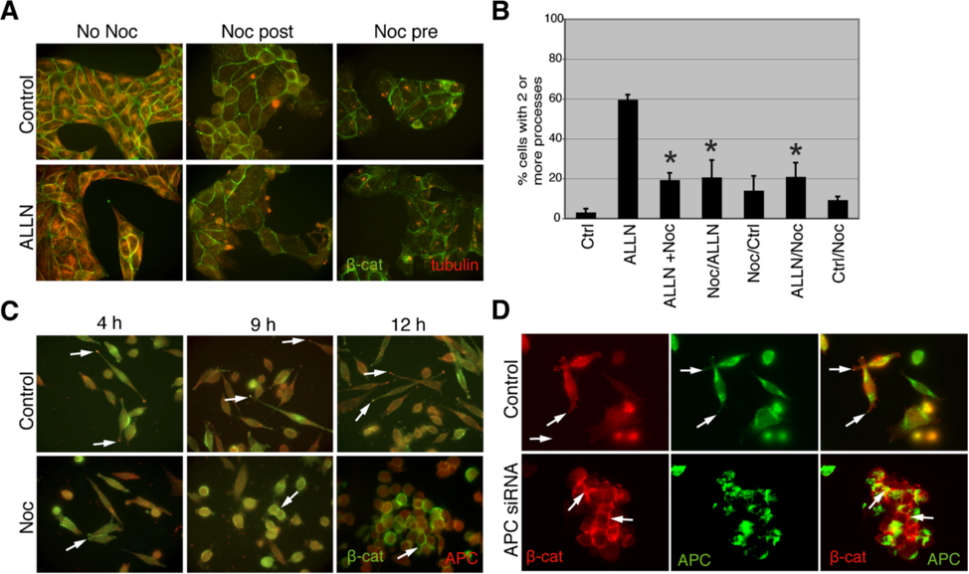
**APC/β-catenin-rich complexes at protrusion ends are dependent on the microtubule cytoskeleton.** (**A**) MDCK cells were treated with 10 μg/ml nocodazole, or DMSO, in combination with 15 μM ALLN and stained with anti-β-catenin (green) and -tubulin (red) antibodies. Microtubule depolymerization inhibited protrusion formation by ALLN in nocodazole (Noc) post- and pre-treated cells compared to controls (No Noc). 630X. (**B**) Quantification shows that protrusions are inhibited by nocodazole treatment, regardless of whether nocodazole and ALLN were added simultaneously (ALLN+Noc), or ALLN was added first (ALLN/Noc) or second (Noc/ALLN) (*p < 0.05). (**C**) 4T07 cells were treated with 10 μg/ml nocodazole or DMSO (control) for 4, 9 or 12 h and stained with antibodies against β-catenin (green) and APC (red). Arrows illustrate punctate APC and β-catenin localization at protrusion ends in control cells. Nocodazole-treated cells lack this localization but demonstrate β-catenin at cell-cell contacts (arrows) that increases as the morphology becomes more epithelial-like over the time course. 630X. (**D**) 4T07 cells were transiently transfected with scrambled (control) or APC-specific siRNAs and stained with anti-β-catenin (red) and –APC antibodies (green). Control cells demonstrate APC and β-catenin co-localization at protrusion ends (arrows), while APC siRNA-transfected cells have a more rounded morphology and increased β-catenin localization at cell-cell contacts (arrows). Images were taken with the same exposure level for APC; cytosolic staining is non-specific. 630x.

Given that APC associates with the plus ends of microtubules, binds to other plus end-binding proteins, and its localization to cortical membrane clusters is microtubule-dependent [10,21,23-27], we next determined whether protrusion formation and APC/β-catenin complexes required intact microtubules. Incubation of MDCK cells in nocodazole attenuated the ALLN-induced phenotype regardless of the order of ALLN and nocodazole treatment (Figure 4A), and quantification of protrusions confirmed these observations (Figure 4B). Importantly, treatment of 4T07 cells with nocodazole over a 4–12 h time frame inhibited the localization of both APC and β-catenin at protrusion ends by 4 h and significantly abrogated protrusion formation by 9–12 h (Figure 4C). Additionally, disruption of microtubules in these cells promoted cell-cell interactions and β-catenin localization to the cell-cell contacts (Figure 4C, arrows). In fact, this phenotype observed with nocodazole treatment was reminiscent of the dramatic change in morphology and β-catenin distribution that we observed with acute knockdown of APC expression using transient expression of APC-specific siRNAs in 4T07 cells (Figure 4D). It should be noted that background staining is frequently observed in APC-knockdown cells upon staining with an anti-APC antibody which could reflect non-specific immunoreactivity of the antibody or residual endogenous APC expression, although the APC localization at protrusion-associated puncta is consistently absent upon APC silencing. Collectively, these data suggest that both APC and intact microtubules are required for β-catenin localization at protrusion ends, the spindle-shaped, mesenchymal morphology and membrane protrusions that are important for tumor cell migration.

## Discussion

While loss of the APC tumor suppressor has been demonstrated in numerous studies to facilitate tumor initiation by activating Wnt signaling and driving proliferation, a role for APC in later stages of tumor progression, including morphological changes and tumor cell migration, has not been clearly demonstrated. Moreover, the importance of the distinct subcellular pools of β-catenin at the lateral membrane and nucleus in tumor development have been well documented; yet, the presence of exogenous mutant β-catenin at protrusion ends suggests that this may represent a fraction of β-catenin with a distinct activity. In this study, we have described APC/β-catenin-rich clusters at protrusion ends in multiple models, including 1) epithelial cells in which endogenous β-catenin has been stabilized, 2) epithelial cells undergoing EMT and 3) mouse and human breast tumor cell lines, all of which have a mesenchymal morphology with robust membrane protrusions. Using RNA interference via lentiviral-mediated delivery of shRNAs, we demonstrated that APC is required for maintaining β-catenin localization to the protrusion tips and the mesenchymal phenotype of the 4T07 mouse mammary tumor cell line but does not significantly affect proliferation or β-catenin/TCF-dependent transcriptional activation. Importantly, the migratory behavior of these tumor cells was also dependent on APC.

Several previous studies have demonstrated localization of APC to the leading edge or membrane protrusions of migratory non-transformed cells, including subconfluent epithelial cells, fibroblasts, astrocytes, neurons and endothelial cells [10,14,23,27-29]. As far as we are aware, this work is the first to address the role of these protrusion-associated APC/β-catenin complexes in tumor cells. In these other contexts, APC localizes to the plus-ends of microtubules and the localization of APC to membrane protrusions is microtubule- but not actin-dependent [14,15,25]. This association of APC with microtubule plus ends involves a direct interaction of APC’s carboxy-terminal basic domain with tubulin and indirect association through the plus-end microtubule binding protein, EB1, and the KAP3/Kif3 plus-end-directed motor proteins [30-32]. Recent studies suggest that microtubule-associated APC transports RNA molecules to protrusion ends in fibroblasts [33]. Our observation that APC suppression reduces tumor cell migration is consistent with functional studies indicating that APC is required for directed cell motility in astrocytes [14,15], as well as protrusion formation and wound filling in fibroblasts and osteosarcoma cells [11]. In the latter model systems, this activity for APC was linked to changes in microtubule stability [11]. These data are consistent with our observations in mammary tumor cells that microtubule integrity is required for membrane protrusions driven by APC/β-catenin-complexes and that APC suppression mimics the morphological defects induced by the microtubule de-stabilizing drug, nocodazole. Because of limitations of our work that include primarily providing functional data with one tumor cell line and characterizing *in vitro* tumor cell behavior, it will be important to extend these observations to other tumor models and *in viv*o studies to more fully dissect APC molecular action in cell motility.

*In vivo* studies have also demonstrated that APC loss or mutation impedes enterocyte migration along the crypt-villus axis [34,35] and forced overexpression of APC results in disordered, non-adhesive intestinal cell migration, presumably by changing the relative activity of cadherin-catenin complexes [36]. Additional support for our observation that APC controls cell-cell interactions is provided by studies in APC-mutant colorectal cancer cells in which APC re-introduction induced β-catenin/E-cadherin localization at the adherens junction and promoted a more epithelial, less motile phenotype [37]. It is likely that inconsistencies in the net impact on migration with APC loss or overexpression between these models and *in vitro* studies are due to cell-type specific activities, analyzing specific *APC* mutations vs. loss vs. ectopic expression, differences between studying normal and tumor cells, and the contribution of tissue architecture and substratum in guiding epithelial cell migration. It is also interesting that our 4T07 cells with the greatest extent of APC knockdown (APC shRNA1) had a more modest effect on migration but slightly more robust impact on morphology. Because these are antibiotic-resistant pools upon lentiviral infection, the populations are heterogeneous and may have various levels of APC from cell to cell at a wound edge for example, a concern could be mitigated by subcloning. It is also possible that these differences reflect a biologically significant threshold or local concentration of APC necessary for protrusion formation, cell motility and cell-cell interactions. However, we are confident in the specificity of the knockdown with both shRNAs because the same phenotypes are observed with multiple, independent shRNAs, and APC expression and defects in other cell lines associated with these constructs have been rescued with ectopic expression of a silencing-resistant human full-length APC cDNA (JRP and KHG, manuscript in preparation).

The data presented here indicate that the observed effects of APC depletion are independent of Wnt signaling activation, since β-catenin/TCF transcriptional activity is not detected in APC-knockdown 4T07 cells. Despite the fact that Wnt signaling has been implicated in promoting EMT and tumor cell motility and invasion [38-41], our data support a separation of these activities in this model system. Additionally, these Wnt-independent effects of APC in 4T07 mammary tumor cells are consistent with our previous characterization of APC-mediated regulation of epithelial polarization and tissue architecture and tumorigenesis in the mammary gland [5,6] that do not involve Wnt pathway hyperactivation, suggesting that Wnt-independent APC activities may be context- or tissue-specific. We cannot rule out that there is redundant action of APC2, for example, in the APC-knockdown cells sufficient for restricting Wnt pathway activation. In any case, a direct effect of APC on migration or invasion may reflect that its regulation of membrane protrusions is required for both migration and invasion or may involve the association of APC with the actin cytoskeleton, for example. Unfortunately, we were unable to separate a contribution of APC specifically to tumor cell invasion from its impact on motility (M.O and K.H.G., unpublished observations). Although APC localization to protrusion ends is not actin-dependent, APC binds directly to actin and promotes its nucleation [42,43] and alters actin dynamics through its interactions with IQGAP, an effector of Rac and Cdc42 [13], Asef1 and Asef2, Rac-specific guanine-exchange factors [44-46] and mDia, a Rho GTPase effector [27]. Moreover, recent data from our lab and others demonstrate that *APC* mutation result in dramatic changes in cell-matrix signaling pathways, including focal adhesion kinase (FAK) and Src [6,47]. Collectively, these findings suggest that APC inactivation in tumor cells may elicit widespread changes in cytoskeletal dynamics and integrin signaling to modify tumor cell behavior.

Our results demonstrating that stabilized endogenous β-catenin localizes to APC clusters at membrane extensions are generally consistent with studies in which an exogenous stabilized mutant β-catenin localized to MDCK protrusions [20] but also extends these findings to tumor models. Recent studies indicated that, in MDCK cells, phosphorylation of β-catenin at its amino-terminus leads to its localization to cell protrusion ends as well as adherens junctions and that the pool at protrusion ends associates with APC and is phosphorylated by GSK3β but is not degraded [48]. Phosphorylated β-catenin is also induced by proteasome inhibition in 293T cells [49]. Also consistent with our results, Faux et al. demonstrated that protrusion-associated β-catenin complexes required APC [48]. In endothelial cells, phosphorylation of APC by GSK3β and CK1 is required for APC and phosphorylated β-catenin to be associated with microtubules in clusters at protrusion ends and for cell migration [28]. Moreover, these clusters are thought to have a high-rate of turnover via the proteasome; whereas a more stable pool of phosphorylated APC and phosphorylated β-catenin is found at the lateral membrane [28], suggesting that complex turnover and post-translation modifications may control the dynamics of distinct APC and β-catenin subcellular pools. Other components of these APC/β-catenin clusters may include the APC binding partners Dlg and Scribble, which have been associated with APC at protrusion ends in astrocytes [15,50] and epithelial cells ([12]; KHG, unpublished results). A detailed analysis to identify all of the complex constituents and the mechanisms that control complex dynamics has not yet been performed, but will be necessary to uncover a clear molecular picture of the membrane-protrusion-associated APC/β-catenin complex in these *in vitro* model systems. The next challenge will be applying this knowledge to elucidate the physiological relevance of these protrusion-associated complexes to normal tissue homeostasis and tumor initiation and progression *in vivo*.

These results also suggest that APC loss late in tumor progression may impact the invasive or metastatic tumor phenotype. Spindle-shaped 4T07 mammary tumor cells are capable of forming primary tumors, intravasating, surviving the blood stream, and extravasating into lungs, but they fail to efficiently colonize to form distant metastases (reviewed in [51]). Depletion of APC in these cells suppressed tumor cell migration but did not significantly impact proliferation. One prediction from these data is that, in contrast to exclusively driving tumor initiation as known to do in colorectal cancer for example, APC loss would actually prevent primary tumor invasion or metastasis to a distant site. However, the consequence of APC inactivation might be dependent on the timing of loss. For example, if APC is mutated or down-regulated after tumor cell extravasation, it may actually promote tumor cell colonization by promoting cell-cell or cell-matrix interactions. Although neither possibility has yet been tested experimentally, recent studies indicate that *APC* gene silencing by methylation is associated with reduced disease-free overall survival in breast cancer patients [52], serum APC levels are associated with colorectal cancer metastases [53] and *APC* somatic mutations and LOH is observed frequently in brain metastases from lung and colorectal primary tumors [54,55]. It will also be important to determine if there are distinct consequences of *APC* mutation and down-regulation on cell motility and tumor progression, which differ in frequency among tumor types. This is likely to be the case because mutant APC appears to have a gain-of-function binding to the Asef proteins that control tumor cell migration [44-46]. Careful comparison of *APC* mutation and silencing in well-defined model systems is required to address this issue. However, our findings support the concept that the timing of APC loss during tumor progression may be important in dictating the biological consequences and suggest that APC, and its downstream effectors, might even represent novel therapeutic targets to modulate tumor metastasis.

## Conclusions

Our study demonstrates that the localization of APC/β-catenin-rich complexes at membrane protrusion ends is required for tumor cell morphology and motility. These data expand our understanding of the consequences of *APC* mutation or down-regulation on tumor cell behavior and suggest that the precise timing of APC loss during tumor progression may dictate whether there is an impact tumor cell dissemination and/or colonization.

## Abbreviations

ALLN: N-Acetyl-Leu-Leu-Nle-CHO; APC: Adenomatous polyposis coli; BrdU: 5-bromo-2'-deoxyuridine; DMSO: Dimethyl sulfoxide; EMT: Epithelial-to-mesenchymal transition; FAK: Focal adhesion kinase; GSK3β: Glycogen synthase kinase 3 beta; IF: Immunofluorescence; LOH: Loss of heterozygosity; MDCK: Madin-Darby Canine Kidney; qRT-PCR: Quantitative reverse-transcriptase polymerase chain reaction; shRNA: Short-hairpin RNA; siRNA: Small interfering RNA; TCF: T cell factor.

## Competing interests

All authors declare that we have no competing interests.

## Authors’ contributions

The study was conceived by KHG. JRP generated the stable APC-knockdown cell lines and performed the reporter, siRNA experiments and nocodozole studies. MAO performed the wound-filling, western blotting, BrdU assays and some IF studies. KHG performed many of the IF analyses. The manuscript was written by MAO, JRP and KHG, and all authors read and approved the final manuscript.

## Pre-publication history

The pre-publication history for this paper can be accessed here:

http://www.biomedcentral.com/1471-2407/13/12/prepub
